# Case Report: Unmasking an anomalous coronary artery during emergency STEMI intervention: a rare diagnostic challenge with successful revascularization outcome

**DOI:** 10.3389/fcvm.2026.1909150

**Published:** 2026-08-31

**Authors:** Muhammad Saad Reihan, Tarek Ahmed Ahmed Dabash, Ahmed Mohammed Ahmed Mostafa, Mohammed Ali Mohammed Hammad, Husna Irfan Thalib, Hamdy Abdelazeem Elkafory, Mohammed Moanes Mohammed Mohyeldin

**Affiliations:** 1 Cardiology Department, Faculty of Medicine, Al-Azhar University, New Damietta, Egypt; 2 Department of Internal Medicine, General Medicine Practice Program, Batterjee Medical College, Jeddah, Saudi Arabia; 3 Hayat National Hospitals, Riyadh, Saudi Arabia; 4 Cardiology Department, Faculty of Medicine, Kafr Elsheikh University, Kafr Elsheikh, Egypt; 5 General Medicine Practice Program, Batterjee Medical College, Jeddah, Saudi Arabia; 6 Cardiology Department, Faculty of Medicine, Al-Azhar University, Cairo, Egypt; 7 Hayat National Hospitals, Jazan, Saudi Arabia

**Keywords:** anomalous right coronary artery, coronary artery anomaly, left coronary cusp, primary percutaneous coronary intervention, ST-elevation myocardial infarction

## Abstract

**Background:**

Coronary artery anomalies during primary PCI are uncommon but can increase procedural complexity and delay reperfusion in STEMI. Anomalous origin of the right coronary artery (RCA) from the left coronary cusp is a rare congenital variant that poses diagnostic and technical challenges in emergency intervention.

**Case presentation:**

A 39-year-old female with insulin-dependent diabetes presented with 10-hour chest pain, diaphoresis, and dyspnea. She was in acute pulmonary edema (Killip III) with sinus tachycardia. ECG showed extensive anterior STEMI, and troponin exceeded 50,000 ng/L. Echocardiography revealed LVEF 39% with apical hypokinesia. She received standard STEMI therapy and underwent urgent PCI. Radial access failed due to spasm, requiring femoral crossover. Angiography showed proximal LAD occlusion (TIMI 0) and difficulty engaging the RCA. Non-selective aortic cusp injection revealed anomalous RCA origin from the left coronary cusp. Primary PCI to the LAD was performed with a drug-eluting stent, restoring TIMI 3 flow. Door-to-wire time was 52 min and fluoroscopy time 11.5 min. The patient improved clinically, with troponin reduction to 26,000 ng/L and discharge after 5 days. At 1-month follow-up, LVEF improved to 45%.

**Conclusion:**

Anomalous RCA origin from the left coronary cusp is a rare but critical finding in STEMI PCI. Early recognition and adaptive catheter strategy are essential to avoid delay and ensure successful reperfusion.

## Introduction

Coronary artery anomalies are uncommon congenital abnormalities with a reported prevalence ranging from 0.3% to 1.3% in patients undergoing coronary angiography. Among these anomalies, anomalous origin of the right coronary artery (RCA) from the left coronary cusp is considered a rare entity that may create substantial diagnostic and technical challenges during coronary angiography and emergency percutaneous coronary intervention (PCI) (1–3).

Primary PCI remains the standard reperfusion strategy for acute ST-elevation myocardial infarction (STEMI), where rapid restoration of coronary blood flow is essential to reduce myocardial damage and improve clinical outcomes. However, the presence of anomalous coronary anatomy may increase procedural complexity, prolong reperfusion time, and complicate catheter engagement during emergency intervention (4).

Recognition of coronary anomalies during primary PCI is therefore critically important, particularly in unstable patients presenting with heart failure or cardiogenic shock. Appropriate catheter selection, rapid identification of the anomalous vessel origin, and procedural expertise are essential for successful revascularization while minimizing procedural delay (5).

We report a challenging case of anterior STEMI in a young adult female with long-standing insulin-dependent diabetes mellitus complicated by acute pulmonary edema, where primary PCI revealed an anomalous RCA arising from the left coronary cusp.

## Case presentation

A 39-year-old female with a history of long-standing insulin-dependent diabetes mellitus (IDDM). In addition to a known history of type 1 diabetes mellitus, her cardiovascular risk factor profile was notable for atherogenic dyslipidemia with elevated LDL-C levels. There was no family history of premature ischemic heart disease in first-degree relatives.

**Figure 1:**
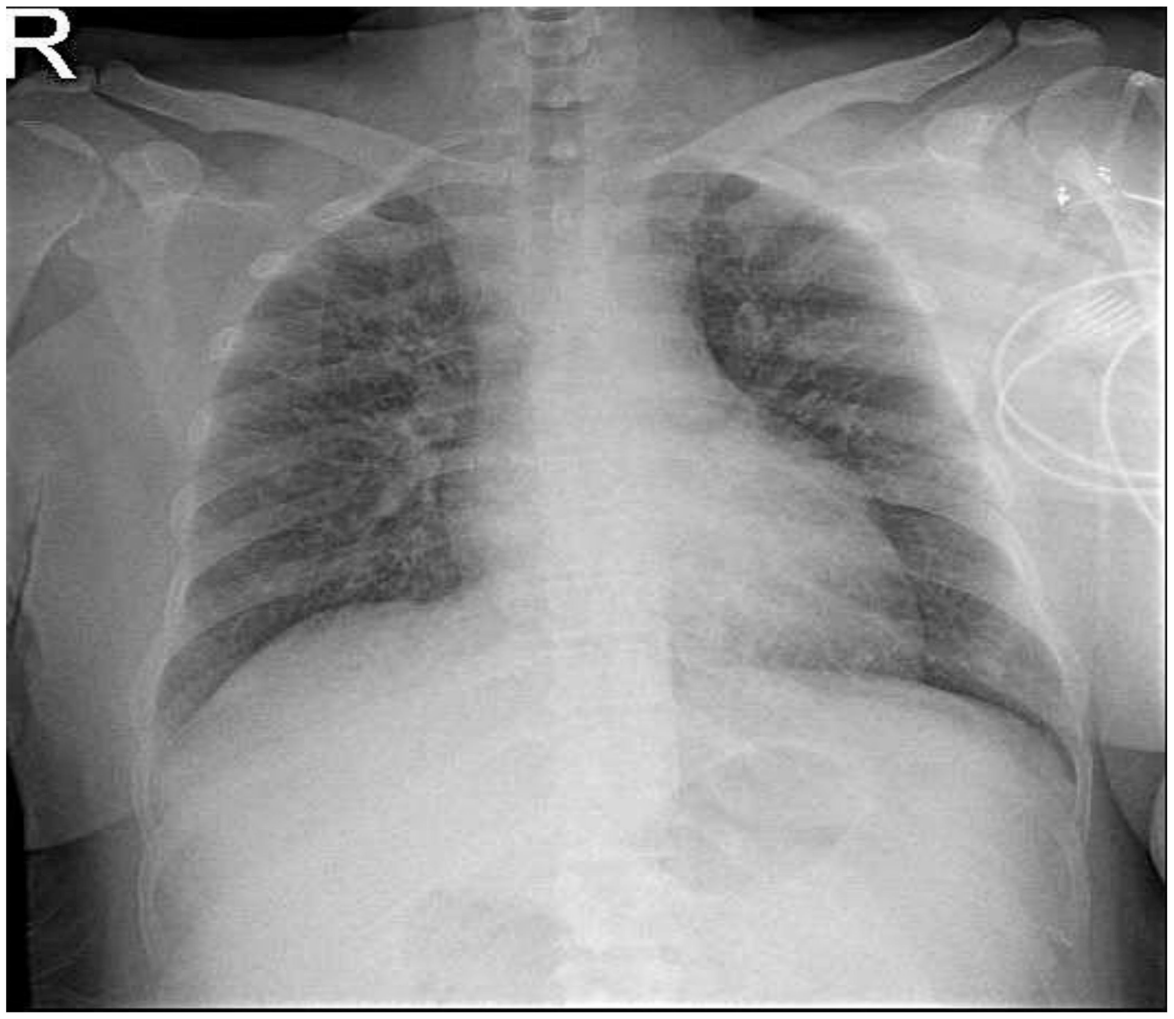
Chest radiography demonstrating bilateral pulmonary congestion and acute pulmonary edema on admission.

She presented to the emergency department with severe typical central chest pain of 10-hour duration associated with nausea, profuse sweating, and progressive dyspnea. On admission, the patient was in respiratory distress and classified as Killip class III due to acute pulmonary edema as as demonstrated on the chest radiograph in Figure 1.

Vital signs revealed blood pressure of 130/80 mmHg and sinus tachycardia with heart rate of 134 beats/minute. Cardiovascular examination demonstrated a galloping rhythm, while chest examination revealed bilateral basal crepitations consistent with pulmonary congestion.

**Figure 2:**
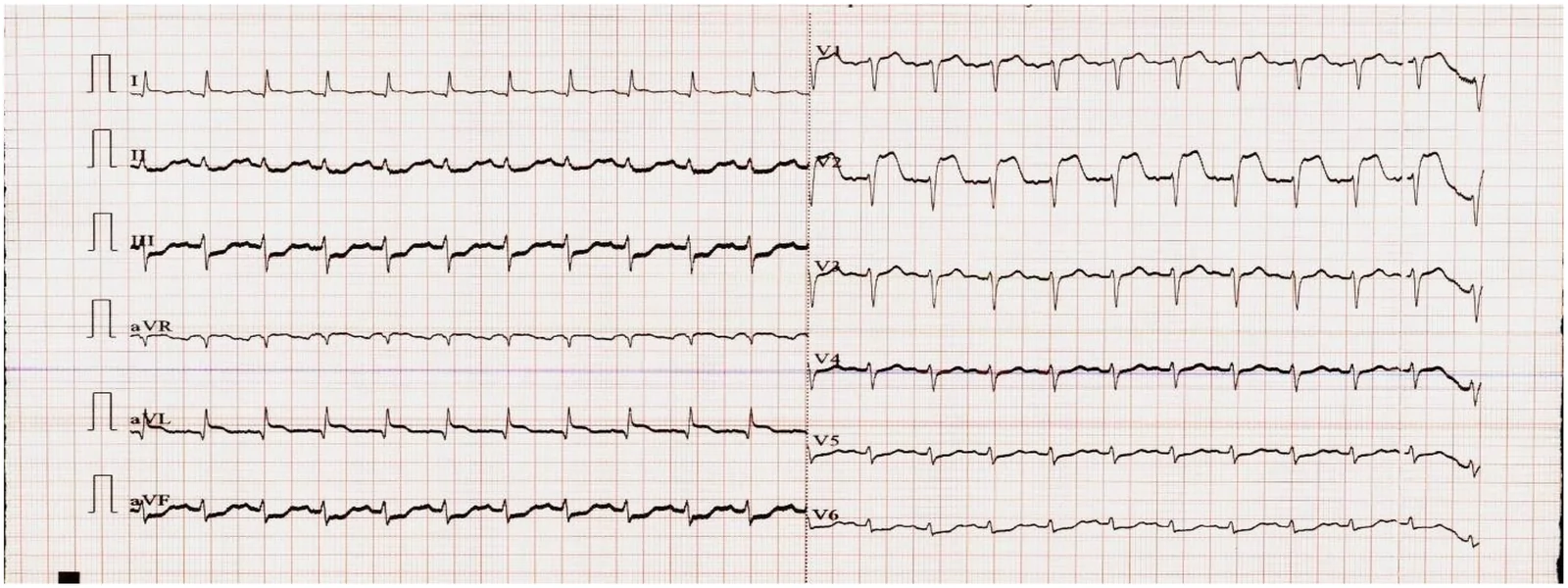
Electrocardiogram on presentation demonstrating ST-segment elevation in leads V1–V3, I, and aVL with reciprocal inferior ST-segment depression consistent with extensive anterior STEMI.

Electrocardiography demonstrated ST-segment elevation in leads V1–V3, I, and aVL with reciprocal ST-segment depression in the inferior leads, consistent with acute extensive anterior STEMI (Figure 2).

Initial high-sensitivity troponin level was markedly elevated (>50,000 ng/L). Bedside transthoracic echocardiography revealed left ventricular ejection fraction (LVEF) of 39%, mild mitral regurgitation, and severely hypokinetic apical segments with preserved myocardial thickness, suggesting viable myocardium.

The patient received emergency anti-ischemic and heart failure management in the emergency department including morphine, aspirin, clopidogrel, intravenous unfractionated heparin, and furosemide, followed by urgent transfer to the cardiac catheterization laboratory for primary PCI with a door-to-wire crossing time of 52 min.

Coronary angiography was initially attempted through right radial access. Following sheath insertion, intra-arterial vasodilators (400 mcg nitroglycerin and 200 mcg verapamil) were administered per standard institutional protocol to prevent radial artery spasm. Despite this prophylactic spasmolytic therapy, severe and refractory radial artery spasm developed, prompting a safe crossover to transfemoral access.

**Figure 3:**
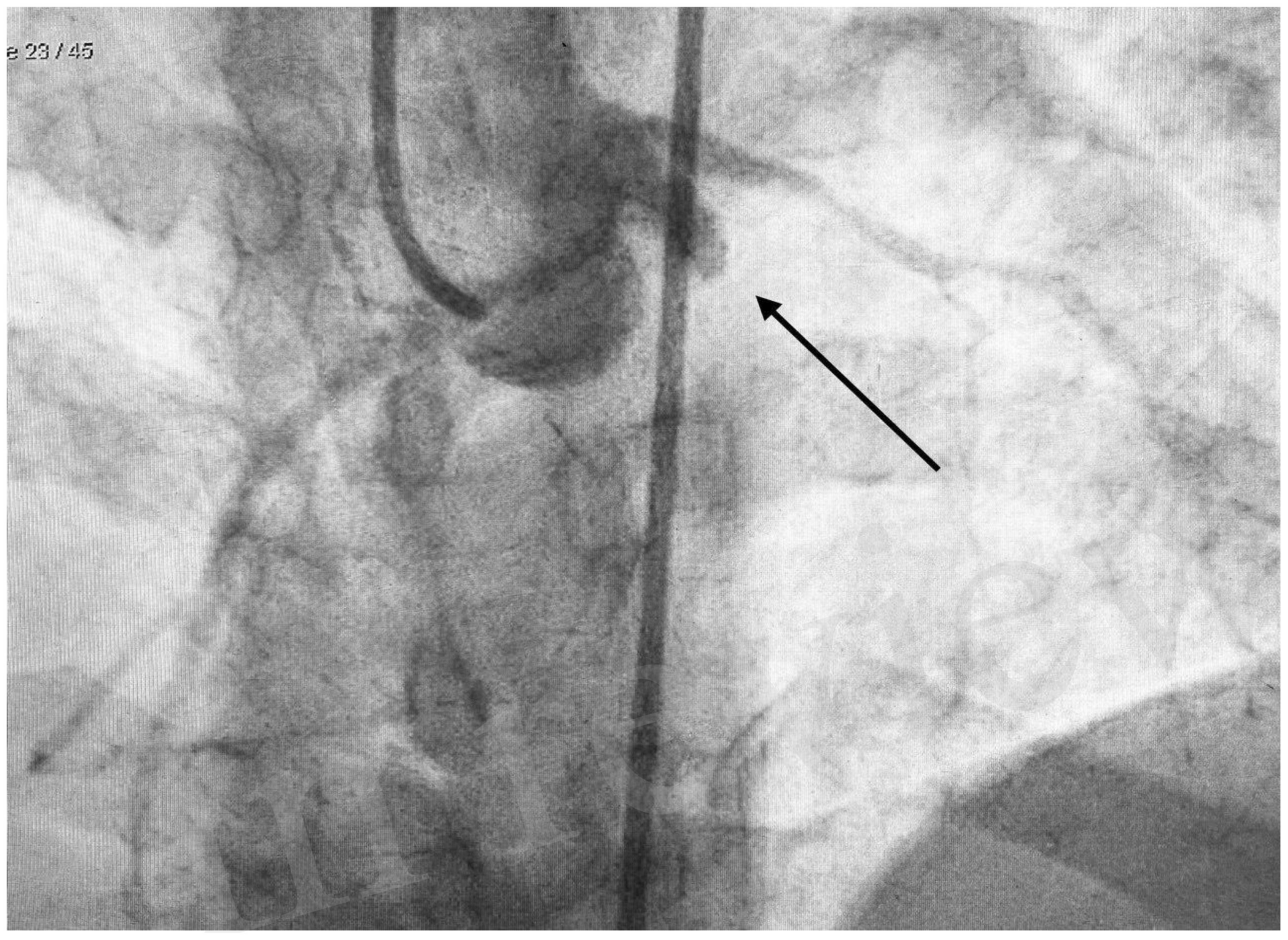
Non-selective cusp angiography demonstrating anomalous origin of the right coronary artery arising from the left coronary cusp with simultaneous visualization of total proximal LAD occlusion (black arrow).

Attempts to selectively cannulate the RCA using a Judkins Right 3.5 catheter at the usual anatomical location were unsuccessful. Subsequent non-selective cusp injection demonstrated anomalous origin of the RCA arising from the left coronary cusp adjacent to the left main coronary artery. Given the clinical urgency of the acute myocardial infarction and the need to minimize door-to-wire crossing time, non-selective angiographic injections were performed near the left coronary sinus using a Judkins Right 3.5 (JR 3.5) catheter. This approach successfully delineated the anomalous RCA origin from the left sinus of Valsalva without prolonging procedural time with multiple catheter exchanges (Figure 3).

Further non-invasive imaging, such as coronary computed tomography angiography (CCTA), was recommended to delineate the vessel trajectory; however, the patient refused further diagnostic workup. Consequently, the precise anatomical course of the anomalous RCA whether interarterial, retroaortic, prepulmonic, or subpulmonic could not be definitively characterized.

**Figure 4:**
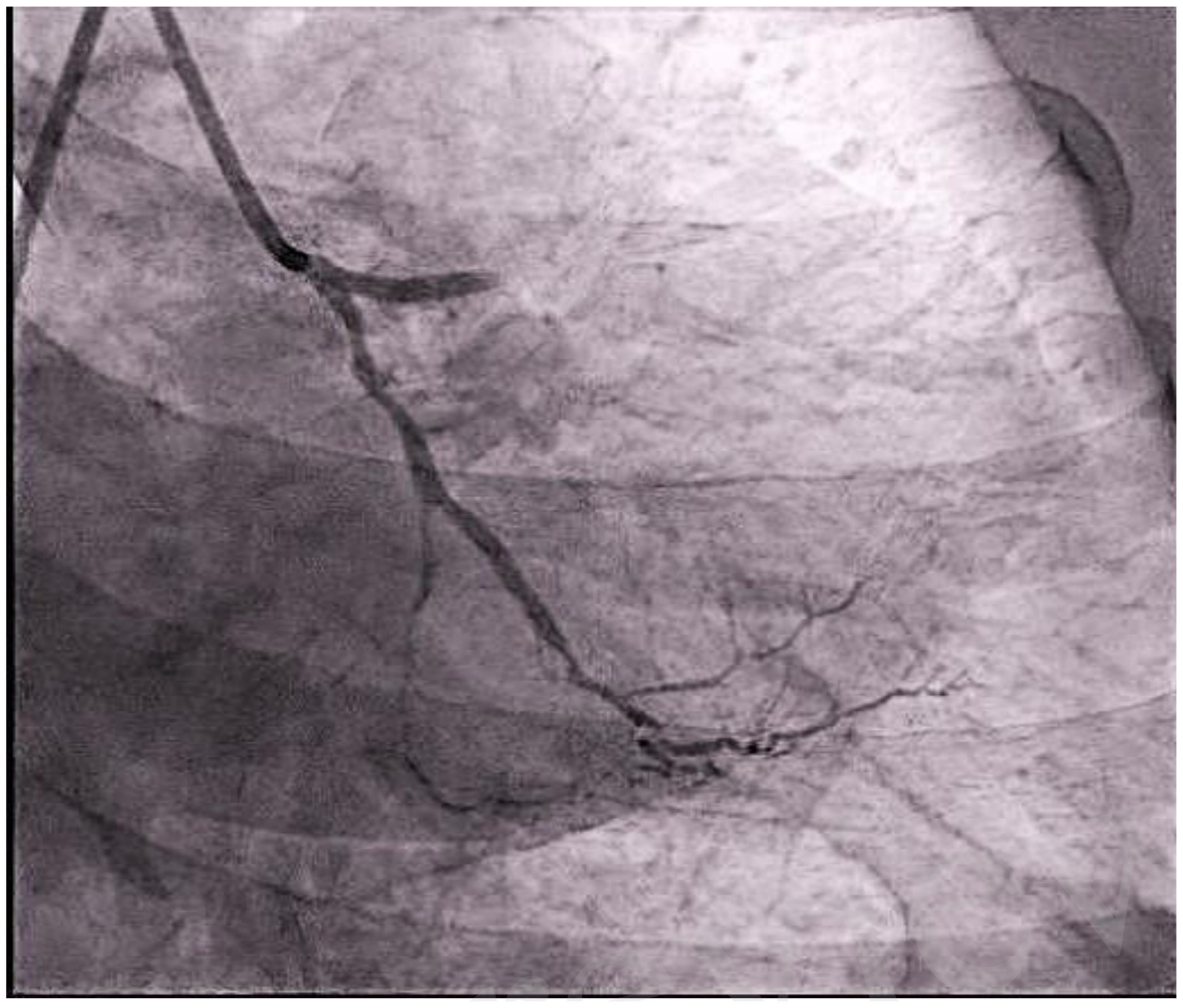
Selective left coronary angiography in right anterior oblique caudal projection demonstrating total proximal LAD occlusion with TIMI 0 flow.

Left coronary angiography via a JL 3.5 guiding catheter demonstrated a total proximal occlusion of the left anterior descending artery (LAD) with TIMI 0 flow, an estimated lesion length of 12–14 mm, and a Grade 5 thrombus burden. Concomitant non-obstructive atherosclerotic disease was noted in the left circumflex artery (LCX) (Figure 4).

**Figure 5:**
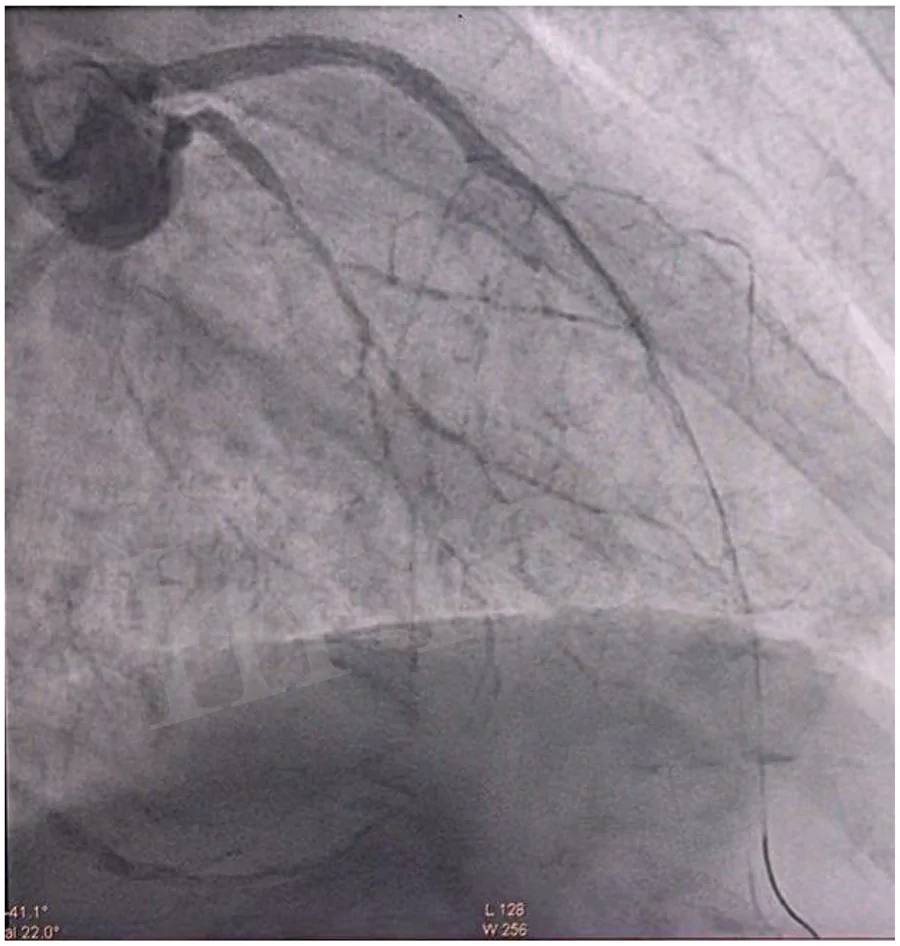
Final coronary angiography in right anterior oblique cranial projection demonstrating successful LAD revascularization with restoration of TIMI 3 flow after stent implantation.

Primary PCI to the LAD was performed using a Soft SWL Royal guidewire followed by predilatation with a 2.0 × 15 mm DEMAX compliant balloon. A 3.0 × 15 mm Resolute Onyx Frontier drug-eluting stent was deployed at 14 atmospheres followed by post-dilatation using a 3.5 × 10 mm non-compliant DEMAX balloon with successful restoration of TIMI 3 flow (Figure 5).

Door-to-wire crossing time was 52 min with total procedural duration of 23 min. Fluoroscopy time was 11 min and 35 s with total contrast volume of 120 mL.

**Figure 6:**
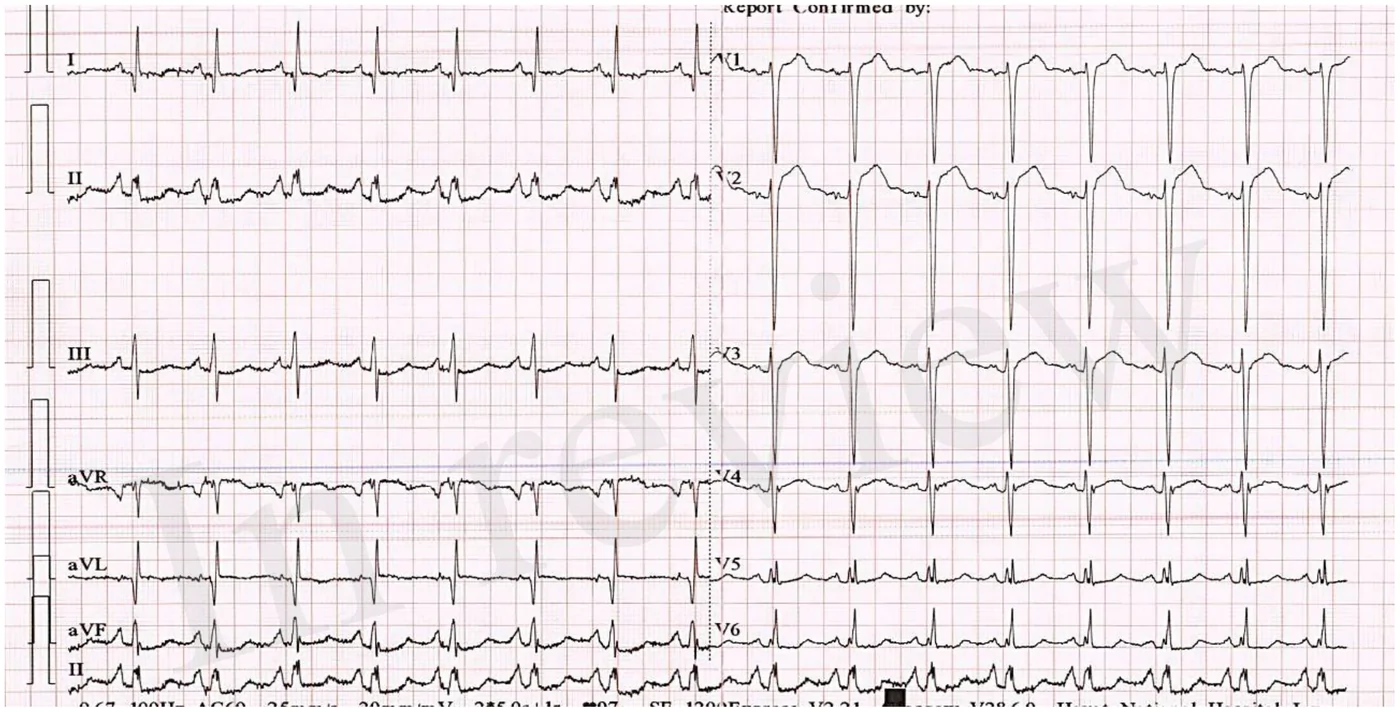
Electrocardiogram after successful revascularization and prior to hospital discharge demonstrating significant ST-segment resolution.

The patient demonstrated progressive clinical improvement with resolution of pulmonary edema and significant ST-segment resolution in predischarge ECG (Figure 6). High-sensitivity troponin level decreased from >50,000 ng/L to 26,000 ng/L prior to discharge. Total hospital stay was 5 days including 3 days in the intensive care unit.

The patient was discharged on dual antiplatelet therapy (DAPT) alongside guideline-directed medical therapy for heart failure and myocardial ischemia. At the one-month follow-up, the patient remained in stable clinical condition with NYHA Class I–II symptoms, demonstrating no features of acute heart failure decompensation or recurrent ischemic events. Follow-up transthoracic echocardiography demonstrated significant improvement in left ventricular systolic function, with an LVEF recovering to 45%. Although the patient initially reported mild dyspnea, this was clinically attributed to ticagrelor-induced dyspnea; symptoms resolved dramatically following a transition to clopidogrel with no adverse ischemic or thrombotic events. In accordance with current clinical consensus guidelines on antiplatelet switching, a 600 mg loading dose of clopidogrel was administered 24 h after the final 90 mg dose of ticagrelor, followed by a daily maintenance dose of 75 mg.

## Discussion

Coronary artery anomalies are uncommon findings during coronary angiography and are frequently discovered incidentally. Anomalous origin of the RCA from the left coronary cusp represents one of the clinically significant coronary anomalies due to its potential association with myocardial ischemia, sudden cardiac death, and technical challenges during coronary intervention (1–3).

Primary PCI in STEMI patients with anomalous coronary anatomy presents a unique procedural challenge because rapid reperfusion remains critically important for myocardial salvage. Difficulty in engaging the anomalous vessel may prolong procedural and fluoroscopy times, potentially delaying reperfusion in unstable patients (4).

In the present case, the patient presented after a prolonged ischemic delay of approximately 10 h from symptom onset, primarily attributable to delayed health-seeking behavior and the patient's initial underestimation of symptom severity, resulting in failure to seek immediate medical attention. This clinically significant delay likely contributed to the severity of presentation, including extensive anterior STEMI, acute pulmonary edema (Killip class III), and impaired left ventricular systolic function. Prolonged total ischemic time is strongly associated with larger infarct size, increased microvascular obstruction, higher risk of heart failure, and increased short- and long-term mortality despite successful reperfusion therapy. In the present case, delayed presentation may have contributed to the markedly elevated high-sensitivity troponin level (>50,000 ng/L), reduced LVEF (39%), and extensive regional wall-motion abnormalities observed on echocardiography. This case underscores the importance of public awareness, early symptom recognition, and prompt medical evaluation in STEMI, particularly among young diabetic patients who may underestimate the seriousness of ischemic chest pain and present late after symptom onset. The coexistence of delayed anterior STEMI presentation, acute pulmonary edema, severe left ventricular dysfunction, and anomalous RCA anatomy substantially increased procedural complexity. Initial inability to engage the RCA using standard Judkins catheter raised suspicion of anomalous coronary anatomy, which was subsequently confirmed by non-selective cusp injection.

Recognition of anomalous coronary anatomy during emergency PCI requires a high index of suspicion, especially when standard catheter engagement fails. Non-selective aortic root or cusp angiography plays an essential role in identifying the anomalous coronary origin and guiding subsequent catheter selection (5).

Table 1 summarizes previously reported cases involving an anomalous RCA arising from the left coronary cusp during primary PCI.

**Table 1:** Previously reported cases of anomalous RCA origin from the left coronary cusp encountered during primary PCI (6–9).

Author/Year	Clinical presentation	Coronary anomaly	PCI challenge	Management	Key learning point
Kumar et al. 2023 (6)	Recurrent chest pain with ventricular arrhythmia	RCA arising from left coronary cusp with interarterial course	Difficulty in diagnostic interpretation and catheter engagement	Conservative management	Recognition of anomalous RCA is important for risk stratification and ischemia assessment
Rawala et al. 2019 (7)	Ischemic chest pain without obstructive coronary disease	RCA originating from left coronary cusp	Angiographic identification challenge	Surgical referral	Anomalous RCA may cause ischemic symptoms even without obstructive CAD
Azhari et al. 2025 (8)	STEMI requiring emergent PCI	Anomalous RCA from left coronary cusp	Difficulty engaging anomalous ostium during primary PCI	Successful PCI using tailored guiding catheter strategy	Early identification of anomalous anatomy may reduce reperfusion delay and procedural complications
Shavazi et al. 2025 (9)	STEMI requiring primary PCI	Single ostial origin of the entire coronary circulation from the right coronary sinus	Identification and engagement of an unusual single coronary ostium during emergency PCI	Successful primary PCI using an anatomy-adapted interventional strategy	Prompt recognition of unusual coronary anatomy and flexible catheter selection are essential to avoid reperfusion delay

A recently reported case by Shavazi et al. described STEMI in a patient with a single coronary ostium arising from the right coronary sinus, requiring primary PCI in the presence of markedly abnormal coronary anatomy. Although both cases demonstrate the importance of promptly recognizing an unexpected coronary origin during emergency intervention, the anatomical abnormalities and procedural approaches differed. In the case reported by Shavazi et al., the entire coronary circulation originated from a single right-sided ostium, whereas the present patient had an anomalous RCA arising from the left coronary cusp with a normally originating left coronary system. In our case, non-selective cusp angiography using a JR 3.5 catheter allowed rapid identification of the anomalous RCA, while a JL 3.5 guiding catheter was used for successful PCI of the culprit LAD lesion. Together, these cases emphasize the importance of procedural flexibility, selective use of non-standard angiographic projections, and appropriate catheter selection when unexpected coronary anatomy is encountered during primary PCI (9).

Several catheter strategies have been described for anomalous RCA cannulation arising from the left coronary cusp including Judkins Left, Amplatz Left, multipurpose, and extra backup guiding catheters (10). In our case, non-selective injections near the left coronary sinus using a JR 3.5 catheter enabled identification of the anomalous RCA origin. A JL 3.5 guiding catheter was then used for left coronary angiography and PCI of the culprit proximal LAD lesion.

Despite prolonged ischemic time due to late presentation, successful reperfusion with restoration of TIMI 3 flow resulted in favorable clinical recovery and partial improvement in left ventricular systolic function at follow-up. This case highlights the importance of rapid recognition of coronary anomalies and procedural adaptability during emergency PCI in STEMI patients.

A key limitation of this case report is the lack of coronary computed tomography angiography (CCTA) to delineate the exact anatomical course (e.g., interarterial vs. prepulmonic or retroaortic) of the anomalous RCA, which carries distinct clinical and therapeutic implications. The patient declined further non-invasive imaging workup after initial evaluation, preventing further characterization of the vessel trajectory.

## Conclusion

Anomalous origin of the RCA from the left coronary cusp is a rare congenital coronary anomaly that may complicate primary PCI in STEMI patients. Early recognition of abnormal coronary anatomy, appropriate catheter selection, and procedural expertise are essential to achieve successful reperfusion while minimizing procedural delay. Emergency coronary intervention in such anatomically challenging situations remains feasible with favorable clinical outcomes when promptly managed.

### Learning points

- Coronary artery anomalies should be suspected during primary PCI when standard catheter engagement is unsuccessful, particularly in emergency STEMI settings.
- Anomalous origin of the right coronary artery from the left coronary cusp may significantly increase procedural complexity and reperfusion delay during primary PCI.
- Non-selective cusp or aortic root angiography is a valuable diagnostic maneuver for rapid identification of anomalous coronary anatomy.
- Prompt recognition of abnormal coronary origin and appropriate catheter selection are essential to achieve successful reperfusion while minimizing ischemic time in STEMI patients.
- Primary PCI in patients with complex coronary anomalies remains feasible with favorable clinical outcomes when managed with procedural adaptability and timely intervention.

## Data Availability

The original contributions presented in the study are included in the article/Supplementary Material, further inquiries can be directed to the corresponding authors.
